# CT-perfusion in peripheral arterial disease – Correlation with angiographic and hemodynamic parameters

**DOI:** 10.1371/journal.pone.0223066

**Published:** 2019-09-27

**Authors:** Bert-Ram Sah, Patrick Veit-Haibach, Klaus Strobel, Martin Banyai, Martin W. Huellner

**Affiliations:** 1 Department of Diagnostic, Interventional, and Pediatric Radiology, Inselspital, University of Bern, Bern, Switzerland; 2 Department of Cancer Imaging, King’s College London, London, England, United Kingdom; 3 Department of Nuclear Medicine, University Hospital Zurich, Zurich, Switzerland; 4 Department of Radiology, University Hospital Zurich, Zurich, Switzerland; 5 University of Zurich, Zurich, Switzerland; 6 Joint Department of Medical Imaging, University of Toronto, Toronto, Canada; 7 Department of Radiology and Nuclear Medicine, Lucerne Cantonal Hospital, Lucerne, Switzerland; 8 Department of Internal Medicine, Subdivision of Angiology, Lucerne Cantonal Hospital, Lucerne, Switzerland; 9 Clinic for Angiology, University Hospital Zurich, Zurich, Switzerland; Universita degli Studi Magna Graecia di Catanzaro, ITALY

## Abstract

**Objective:**

The purpose of this study was the assessment of volumetric CT-perfusion (CTP) of the lower leg musculature in patients with symptomatic peripheral arterial disease (PAD) of the lower extremities, comparing it with established angiographic and hemodynamic parameters.

**Materials and methods:**

Thirty-five consecutive patients with symptomatic PAD of the lower extremities requiring interventional revascularization were assessed prospectively. All patients underwent a CTP scan of the lower leg, and hemodynamic and angiographic assessment. Hemodynamic parameters, specifically ankle-brachial pressure index (ABI), ankle blood pressure (ABP), peak systolic velocity (PSV), and segmental pulse oscillography (SPO) level, were determined. Lesion length and degree of collateralization were assessed by interventional angiography. CTP parameters were calculated with a perfusion software, acting on a no outflow assumption. A sequential two-compartment model was used. Differences in CTP parameters and correlations between CTP, hemodynamic and angiographic parameters were assessed with non-parametric tests.

**Results:**

The cohort consisted of 27 subjects with an occlusion, and eight with a high-grade stenosis. The mean blood flow (BF) was 7.71 ± 2.96 ml/100ml*min^-1^, mean blood volume (BV) 0.71 ± 0.33 ml/100ml, and mean mean transit time (MTT) 7.22 ± 2.66 s. BF and BV were higher in subjects with longer lesions, and BV was higher in subjects with lower ABI. Significant correlations were found between lesion length and BV (*r* = 0.65) and BF (*r* = 0.52). Significant inverse correlations were found between BV and ABI and between BV and ABP (*r* = -0.56, for both correlations).

**Conclusions:**

In our study, we have shown the feasibility of CTP for the assessment of PAD. In the future, this quantitative method might serve as a non-invasive method, possibly complementing the diagnostic workup of patients with peripheral arterial disease.

## Introduction

Peripheral arterial disease (PAD) is one of the major cost-driving factors in public healthcare [1]. Similar to coronary artery disease, it is associated with a history of smoking, diabetes, hypertension, and hyperlipidaemia [2]. One of its predominant clinical substrates and early indicators is pain in the lower extremities, which is caused by ischemia due to insufficient blood flow to the dependent musculature [3]. This may be elicited chronically by progressive luminal narrowing, or acutely by thromboembolic occlusion [4]. Historically, perfusion of the soft tissues of the limbs has been assessed with scintigraphic methods, especially in patients with diabetes [5–8]. Recently, efforts have been made to investigate PAD using computed tomography (CT) angiography, contrast-enhanced magnetic resonance imaging (MRI), and ultrasound (US) [9–11]. To date, there was only little interest in limb perfusion assessment by volumetric CTP [12, 13]. Nevertheless, several potential advantages advocate an evaluation of CT for this task. First, data acquisition by CT is much faster than by MRI, more robust and easily standardizable [12]. Second, clinical CT provides a higher spatial resolution than MRI [14]. Third, in contrast to MRI and US, CT allows for a comparably easy true quantitation of perfusion, with contrast medium (CM) concentrations directly corresponding to attenuation values [15]. Lastly, unlike US, CT is supposedly a non-operator dependant method. However, there are some issues in CTP that need to be addressed. The simultaneous acquisition of large volume data sets, being mandatory for limb assessment, is nowadays possible with a new generation of scanners with a larger *Z*-axis coverage [16]. Beam-hardening artefacts and streak artefacts from adjacent osseous structures may be solved by proper tissue segmentation, volume of interest (VOI) restraints and post-processing algorithms [17]. Radiation exposure is one of the drawbacks of CT imaging, in contrast to US or MRI. However, critical organ doses are not reached with CT perfusion imaging [18]. Furthermore the radiation exposure from perfusion imaging is lower in the extremities compared to chest or abdominal imaging. This is also reflected by considerably different tissue weighting factors in ICRP publication 103 (skin and bone: 0.01, compared to chest or stomach: 0.12) [19]. In contrast to the myocardium and lungs, perfusion imaging of the limbs is less prone to organ motion during scan [20]. Nevertheless, involuntary movement may degrade data acquisition [21]. This is especially true for symptomatic PAD patients suffering from pain. Therefore, the application of motion correction algorithms, as supplied by current processing software, seems to be mandatory [20, 22–24]. Skeletal muscle is not as vascular as myocardium or solid organs such as the liver. Thus, CT contrast enhancement in skeletal muscle is fainter than in these structures. In addition, the venous outflow from skeletal muscles is difficult to assess.

Despite several potential benefits, such as fast and non-invasive quantitation of tissue ischemia, the clinical application of CTP of the limbs has so far been challenged by the aforementioned technical difficulties, and larger patient studies are still missing.

Therefore, the purpose of our study was the assessment of volumetric CTP of the lower leg musculature in patients with symptomatic PAD, and the comparison of CTP parameters with state-of-the-art angiographic and hemodynamic parameters, in order to further characterize the potential role of this non-invasive method for the quantitative in-vivo assessment of limb ischemia.

## Materials and methods

This prospective study was approved by the institutional review board and by the cantonal ethics committee (Kantonale Ethikkommission Luzern). All patients provided informed signed consent prior to the examinations. Thirty-five consecutive patients (median age 72 years, range 48 to 87 years, 13 females, 22 males) with symptomatic PAD of the legs were evaluated. All patients were referred for angioplasty for unilateral interventional revascularization of arteries that provide blood supply to the lower leg. Symptomatic PAD was defined as prevalence of intermittent claudication, ischemic rest pain and ischemic tissue loss such as gangrene or non-healing ischemic ulcers, and ultrasonographic evidence of a hemodynamically significant obstruction of the common iliac, external iliac, superficial femoral or popliteal artery. Medical history was recorded. Further details are given in **S1 File**.

All subjects underwent clinical assessment of hemodynamic parameters within 24 hours prior to revascularization (see below). Angiographic parameters were assessed at the beginning of the interventional revascularization. Immediately before the revascularization procedure, all patients underwent a CTP scan of the lower leg.

### Hemodynamic assessment

Hemodynamic assessment was performed in all patients prior to the planned revascularization. Evidence of hemodynamically significant obstruction was established by non-invasive vascular testing, pulse volume recordings and duplex ultrasound imaging studies.

For the assessment of the maximum ankle-brachial pressure index (ABI) and the systolic ankle blood pressure (ABP; mmHg), patients were placed in supine position at rest for 5–10 minutes in a room with ambient temperature (22–25°C). A digital oscillometric automatic blood pressure device with appropriately sized blood pressure cuffs (HEM-907, Omron, Matsusaka, Japan) was used to measure systolic, diastolic and mean arterial blood pressure on both upper arms simultaneously. Less than 12 mmHg inter-arm systolic pressure gradient was regarded as normal [25]. The ABI was calculated by dividing the highest pressure of each leg by the highest systolic arm pressure. Further details are given in **S1 File**. Arteries in subjects with an ABI ≥ 1.40 were considered incompressible. ABI and ABP values of these subjects were not considered for further analysis, because such values indicate the presence of media sclerosis. An ABI of 1.00 to 1.40 was considered as normal, borderline from 0.91 to 0.99, and pathologic if < 0.91 [26]. The ABI was rated as mildly pathologic from 0.50 to 0.89, and severely pathologic if < 0.49.

Segmental pulse oscillography (SPO) was performed with a two-channel device (Infraton Boucke 3000, Fritac AG, Baden, Switzerland). For this study, only the pulse curves of the lower calf were taken into account. Pulse curves were rated as normal if the wave morphology was dichotomous, mildly pathologic with blunt wave morphology and absence of dichotomy, and severely pathologic with flat waves. Maximum impairment of arterial perfusion in a given segment was characterized by a zero baseline. Ultrasonographic imaging was performed with a clinical ultrasound system (IU 22, Philips, Best, The Netherlands). Clinical standard criteria were applied to quantify the severity of a stenosis. In subjects with vessel stenosis, the ratios of peak systolic velocity (PSV; m/s) in the stenotic segment and prestenotic PSV were calculated.

### CTP assessment

All CTP examinations were performed with a 128-slice CT scanner (Somatom Definition Flash, Siemens Healthcare, Forchheim, Germany) in shuttle mode, allowing for 28.2 cm of axial coverage and 2 s temporal resolution, which included almost all muscles of both lower legs in every subject. After placement on the gantry table, the patients’ feet were fixated using tape to prevent spontaneous movement. The tube current was set to 100 mAs, the tube voltage to 100 kV(p). The duration of the CT-perfusion scan was 60 s, with a rotation time of 2 s. CTP was started 15 s after injection of 60 ml of CM (Ultravist 370, Bayer Healthcare, Leverkusen, Germany) at 5 ml/s. CM was injected into an antecubital vein by a dual-head pump injection device (Stellant D, Medrad, Warrendale, USA), followed by a flush of 50 ml of NaCl at 5 ml/s. The collimation was 64 x 0.6 mm. The reconstruction increment was 10 mm at 10 mm slice width. Image reconstruction was performed with a 512 x 512 pixel matrix and medium smooth B30f kernel. For image post-processing and analysis, the reconstructed images of both legs were transferred to a commercially available computer workstation (Syngo Multimodality Workplace, Siemens Healthcare, Erlangen, Germany).

CTP parameters blood flow (BF; ml/100ml * min^-1^), blood volume (BV; ml/100ml), and mean transit time (MTT; s) were calculated with a perfusion software (Syngo Volume Perfusion CT Body, Siemens Healthcare, Erlangen, Germany), based on the Patlak analysis. Since veins did not show sufficient CM enhancement in the late phase of the dynamic series and since venous enhancement was furthermore often degraded by pathologic flow within varicose veins in our patient cohort, no outflow curve was obtained. Thus, as previously described in the literature, we acted on a no outflow assumption [13, 27–29]. Dataset motion correction and a noise reduction algorithm were applied automatically. Processing thresholds or segmentation tissue limits were -50 HU and +150 HU to exclude bone, vessel wall calcification and other hyperdense material. Window width and center for reference vessel input was +300 HU and +150 HU, respectively. The relative threshold for inside and outside was 50%, an adaptive smoothing filter was used. The vendor’s standard algorithmic parameters were applied. 3D color-coded maps for BF, BV and MTT were generated with a sequential two-compartment model. BF is defined as the amount of blood flowing through 100ml of muscle tissue within one minute. MTT is defined as the average time of contrast agent residence within the muscle tissue. BV is defined as the amount of blood within 100ml of muscle tissue. BV can be expressed as proportion of the total volume of a dedicated voxel. For every patient, an individual arterial input fraction was determined by placing a region of interest (ROI) into the popliteal artery. A dedicated free-hand user-defined ROI was drawn around the calf muscles on every slice and adapted to their confines. Adjacent bones, vessels, and other soft tissue structures were excluded. All image evaluations were performed by two experienced radiologists in consensus with 8 and 12 years of experience.

### Angiographic assessment

Before the start of revascularization, the length of the occlusion or stenosis was measured on subtracted angiographic images (Allura Xper FD, Philips). The lesion length was rated as short (1) if < 4 cm, medium (2) if 4–10 cm, and long (3) if > 10 cm. The degree of collateralization was assessed by visual estimation on angiographic images. It was rated as good (1) if the estimated cross-sectional area of collaterals added up to > 50% of the pre-lesion diameter of the collateralized vessel, medium (2) if < 50%, and poor (3) if only faint or no collaterals were visible. The performing interventional radiologist had 20 years of experience in the field of revascularization procedures.

### Statistical analysis

Demographic, hemodynamic, angiographic and CTP parameters in patients with stenosis and occlusion were compared by Mann-Whitney U-test. Differences of CTP parameters between symptomatic and non-symptomatic limbs were analysed by Wilcoxon signed ranks test. CTP parameters were stratified by lesion length, collateralization, ABI and SPO, and were then compared by Kruskal-Wallis test. The correlation between CTP, hemodynamic and angiographic parameters was analysed using Spearman’s correlation. Results were interpreted as almost perfect correlation between ±0.81 and ±1.00, substantial between ±0.61 and ±0.80, moderate between ±0.41 and ±0.60, fair between ±0.21 and ±0.40, there under no correlation [30]. A p-value of < 0.05 was considered statistically significant. All analyses were performed using IBM SPSS Statistics 21.0.0 (SPSS Inc., Chicago, IL, USA).

## Results

### Patients and clinical parameters

**Fig 1:**
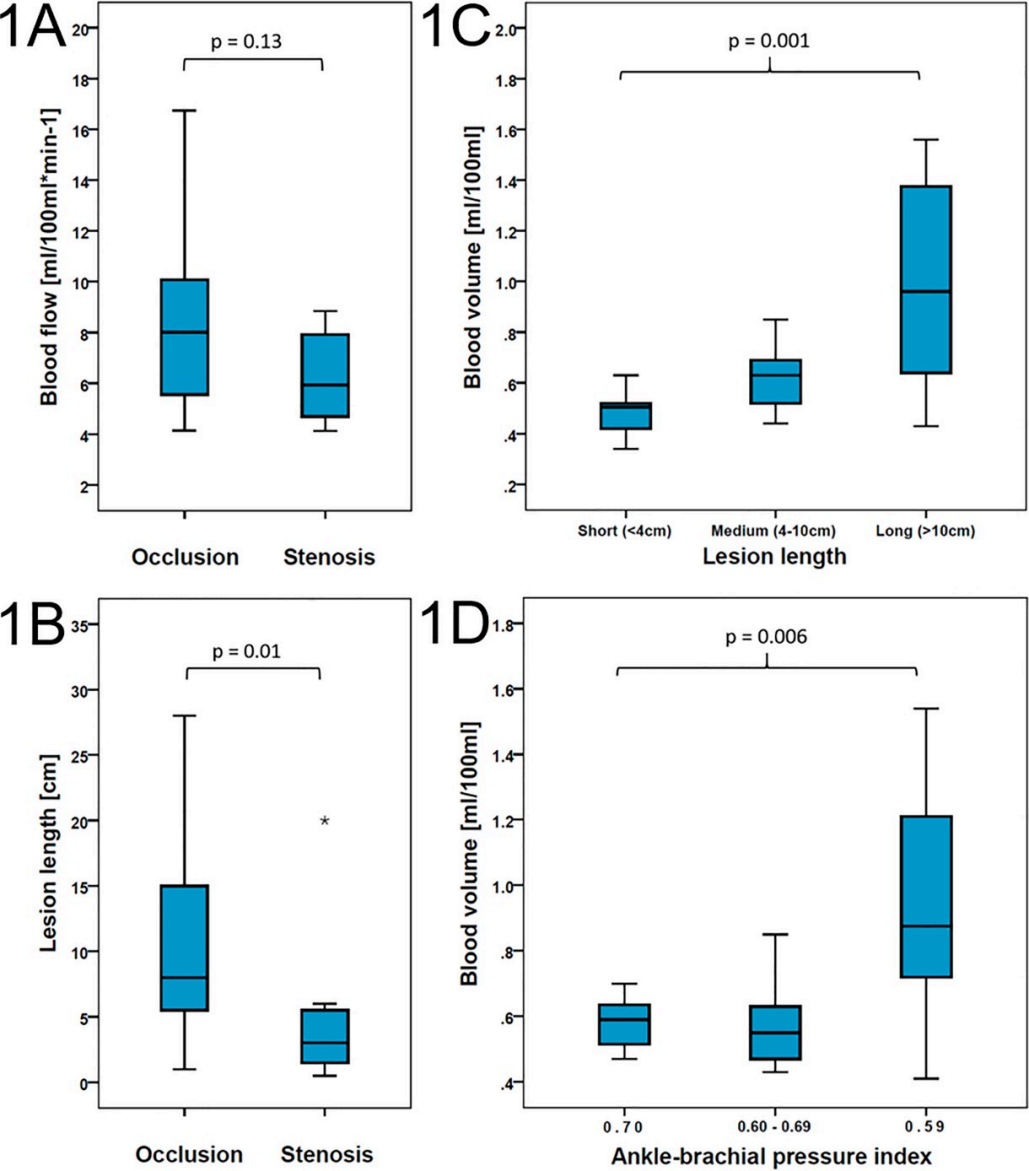
Box plots of CTP parameters and lesion length. **(a)** BF values in patients with occlusion (n = 27) and stenosis (n = 8), p = 0.13. **(b)** Lesion length in patients with occlusion (n = 27) and stenosis (n = 8), p = 0.01. **(c)** BV values in short (n = 10), medium (n = 13) and long lesions (n = 12), p = 0.001. **(d)** BV values in patients with high (n = 8), medium (n = 9) and low (n = 10) ABI values. The upper edge of the box indicates the 75th percentile, the lower edge of the box indicates the 25^th^ percentile, the line within the box indicates the median, and the ends of the whiskers indicate minimum and maximum values. Stars indicate outliers. BF: blood flow, BV: blood volume.

Twenty-seven patients (77%) were referred for interventional revascularization of an occlusion, eight patients (23%) for a high-grade stenosis. All lesions were located unilaterally in the common iliac, external iliac or superficial femoral artery. Occluded vessel segments were significantly longer than stenotic segments (**Fig 1A**), while the degree of collateralization in both types of lesions was not significantly different. Patients with medium and long lesions had a higher degree of collateralization than patients with short lesions, (p = 0.041) (**S1 Table**).

ABP values indicative of media sclerosis were found in 7 subjects (5 occlusions, 2 stenoses). ABP and ABI values of these subjects were hence excluded from analysis. The majority of the remaining patients had mildly pathologic ABI values (n = 22), five had severely pathologic values, and one a normal value. For a comparison of CTP parameters stratified by ABI, subjects were assigned to three equally distributed groups (ABI ≥ 0.70; 0.60–0.69; ≤ 0.59).

PSV could not be determined in 6 subjects (5 occlusions, 1 stenosis). Upstream PSV was significantly higher in stenotic than in occluded vessels. Detailed results are given in **Table 1**.

**Table 1:** Patient data.

Parameter	All (n = 35)	Occlusion (n = 27)	Stenosis (n = 8)	*p-value*
Age [y] (median (range))	72 (48–87)	72 (48–87)	66 (48–85)	0.34
Length of lesion [cm] (mean ± SD)	9.6 ± 7.8	10.9 ± 7.8	5.1 ± 6.3	0.01
Degree of collateralization (median (range))	2 (1–3)	2 (1–3)	2 (2–3)	0.29
ABI (mean ± SD) *	0.62 ± 0.15	0.60 ± 0.14	0.71 ± 0.18	0.19
ABP [mmHg] (mean ± SD) *	86.6 ± 38.0	79.9 ± 23.9	116.0 ± 71.3	0.49
SPO level (median (range))	2 (1–3)	2 (1–3)	2 (1–3)	0.80
PSV [m/s] (mean ± SD) *	1.39 ± 1.74	0.89 ± 1.11	2.98 ± 2.45	0.02
BF [ml/100ml*min^-1^] (mean ± SD)	7.71 ± 2.96	8.14 ± 3.11	6.26 ± 1.85	0.13
BV [ml/100ml] (mean ± SD)	0.71 ± 0.33	0.77 ± 0.35	0.54 ± 0.16	0.07
MTT time [s] (mean ± SD)	7.22 ± 2.66	7.34 ± 2.94	6.80 ± 1.45	0.86

ABI: ankle-brachial pressure index, ABP: ankle blood pressure, BF: blood flow, BV: blood volume, MTT: mean transit time, PSV: peak systolic velocity, SD: standard deviation, SPO: segmental pulse oscillography.

* ABI and ABP values available in 28 patients (22 occlusions, 6 stenoses), PSV values available in 29 patients (22 occlusions, 7 stenoses).

### Absolute CTP values

The mean BF of the symptomatic limb was 7.71 ± 2.96 ml/100ml tissue * min^-1^, the mean BV was 0.71 ± 0.33 ml/100ml tissue, and the mean MTT was 7.22 ± 2.66 seconds. Below we provide data of the asymptomatic limb as well, whenever statistical significance is claimed for CT-parameters in the symptomatic limb, in order to identify potential inter-group differences against intra-individual, inter-limb differences.

### Comparison of CTP parameters in different subgroups

Overall, CTP parameters were neither significantly different in patients with stenosis or occlusion, nor in the symptomatic and asymptomatic limb (**Tables 1 and 2, Fig 1B**). However, in the subgroup with short lesions, higher CTP parameters were found in the asymptomatic than in the symptomatic limb (**Table 2**).

**Table 2:** CTP parameters of the symptomatic and of the asymptomatic limb.

	CT-perfusion parameters	Symptomatic limb	Asymptomatic limb	*p-value*
All lesions (n = 35)	Blood flow [ml/100ml*min^-1^] (mean ± SD)	7.71 ± 2.96	7.75 ± 2.63	0.73
	Blood volume [ml/100ml] (mean ± SD)	0.71 ± 0.33	0.73 ± 0.28	0.77
	Mean transit time [s] (mean ± SD)	7.22 ± 2.66	7.37 ± 2.70	0.58
Short lesions (n = 10)	Blood flow [ml/100ml*min^-1^] (mean ± SD)	5.89 ± 1.35	6.48 ± 1.53	0.05
	Blood volume [ml/100ml] (mean ± SD)	0.49 ± 0.09	0.63 ± 0.18	0.01
	Mean transit time [s] (mean ± SD)	6.19 ± 1.31	6.88 ± 1.77	0.02
Medium lesions (n = 13)	Blood flow [ml/100ml*min^-1^] (mean ± SD)	7.11 ± 1.83	7.26 ± 2.29	0.86
	Blood volume [ml/100ml] (mean ± SD)	0.62 ± 0.13	0.64 ± 0.23	0.51
	Mean transit time [s] (mean ± SD)	7.16 ± 2.54	7.21 ± 2.62	0.35
Long lesions (n = 12)	Blood flow [ml/100ml*min^-1^] (mean ± SD)	9.88 ± 3.66	9.35 ± 3.09	0.37
	Blood volume [ml/100ml] (mean ± SD)	1.00 ± 0.40	0.89 ± 0.32	0.16
	Mean transit time [s] (mean ± SD)	8.13 ± 3.40	7.94 ± 3.46	0.43
ABI ≥0.70 (n = 8)	Blood flow [ml/100ml*min^-1^] (mean ± SD)	7.05 ± 1.44	7.06 ± 1.55	0.89
	Blood volume [ml/100ml] (mean ± SD)	0.58 ± 0.08	0.67 ± 0.26	0.48
	Mean transit time [s] (mean ± SD)	6.71 ± 1.70	7.31 ± 2.07	0.12
ABI 0.60–0.69 (n = 9)	Blood flow [ml/100ml*min^-1^] (mean ± SD)	7.16 ± 2.52	8.05 ± 2.81	0.07
	Blood volume [ml/100ml] (mean ± SD)	0.58 ± 0.13	0.68 ± 0.19	0.24
	Mean transit time [s] (mean ± SD)	6.25 ± 2.00	6.33 ± 2.21	0.77
ABI ≤0.59 (n = 10)	Blood flow [ml/100ml*min^-1^] (mean ± SD)	9.27 ± 3.52	8.01 ± 2.64	0.42
	Blood volume [ml/100ml] (mean ± SD)	0.96 ± 0.35	0.88 ± 0.35	0.19
	Mean transit time [s] (mean ± SD)	8.46 ± 4.06	8.49 ± 4.07	0.96

SD: standard deviation.

BF and BV of the symptomatic leg were significantly different depending on lesion length, with lowest values obtained in short lesions, and highest values in long lesions (**Fig 1C**). For MTT no such difference was found.

CTP parameters were not different when stratified by the degree of collateralization (**Table 3**).

**Table 3:** CTP parameters of the symptomatic limb, stratified by lesion length, degree of collateralization, SPO and ABI.

Groups	n	Blood flow [ml/100ml*min^-1^] (mean ± SD)	*p-value*	Blood volume [ml/100ml] (mean ± SD)	*p-value*	Mean transit time [s] (mean ± SD)	*p-value*
*Length of lesion*							
Short (<4cm)	10	5.89 ± 1.35		0.49 ± 0.09		6.19 ± 1.31	
Medium (4-10cm)	13	7.11 ± 1.83	0.01	0.62 ± 0.13	0.001	7.16 ± 2.54	0.29
Long (>10cm)	12	9.88 ± 3.66		1.00 ± 0.40		8.13 ± 3.40	
*Degree of collateralization*							
Good	11	7.79 ± 2.33		0.74 ± 0.31		7.20 ± 2.75	
Medium	17	7.54 ± 3.57	0.62	0.71 ± 0.36	0.79	7.55 ± 2.96	0.72
Poor	7	8.01 ± 2.53		0.70 ± 0.32		6.44 ± 1.74	
*SPO*							
Normal	4	9.11 ± 3.81		0.72 ± 0.40		5.90 ± 2.50	
Mildly pathologic	20	9.37 ± 7.60	0.48	0.53 ± 0.72	0.70	6.24 ± 7.31	0.66
Severely pathologic	11	7.41 ± 3.80		0.64 ± 0.71		6.53 ± 7.53	
*ABI*							
≥0.70	8	7.05 ± 1.44		0.58 ± 0.08		6.71 ± 1.70	
0.60–0.69	9	7.16 ± 2.52	0.17	0.58 ± 0.13	0.006	6.25 ± 2.00	0.73
≤0.59	10	9.27 ± 3.52		0.96 ± 0.35		8.46 ± 4.06	

ABI: ankle-brachial pressure index, SD: standard deviation, SPO: segmental pulse oscillography.

There was no significant difference in CTP parameters when stratified by the SPO level.

BV of the symptomatic limb was significantly different among ABI groups (*p* = 0.006), with higher BV in patients with lower ABI (**Fig 1D**). In the asymptomatic limb, there was no significant difference of BV among the ABI groups (p = 0.18).

### Correlation of CTP parameters

**Fig 2:**
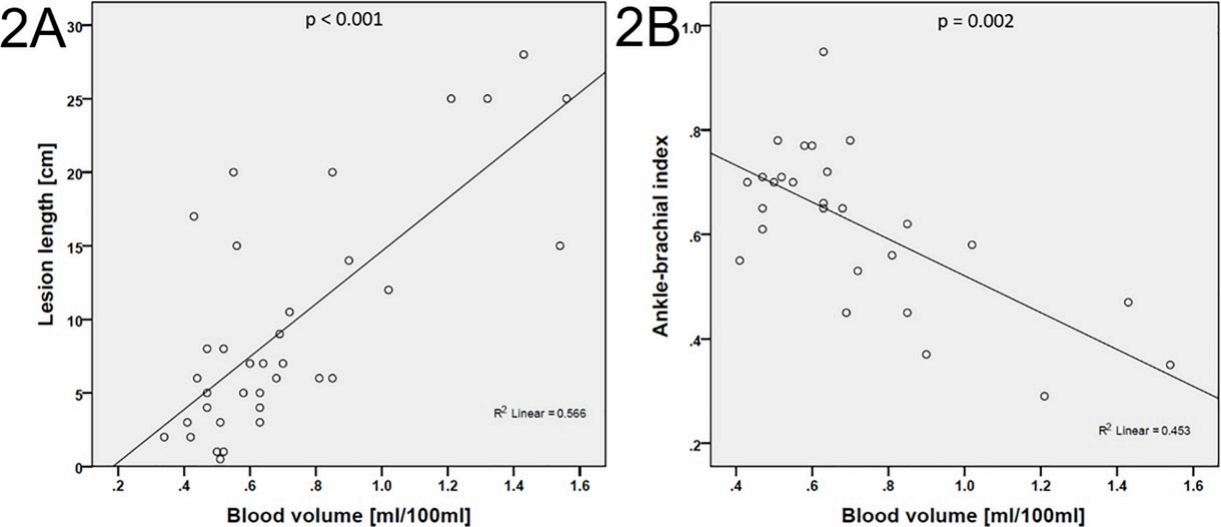
**Scatter plots of BV and lesion length (A) and BV and ABI (B)**. ABI: ankle-brachial pressure index, BV: blood volume.

A substantial correlation was found between BV and lesion length (*r* = 0.65, *p* < 0.001; **Fig 2A**), and a moderate correlation between BF and lesion length (*r* = 0.52, *p* = 0.001). BV showed a moderate inverse correlation with ABI (**Fig 2B**) and ABP (*r* = -0.56, *p* = 0.002, each). Similar correlations were obtained in the subgroup analysis of subjects with occlusions, although by trend with lower coefficients (**Table 4**).

**Table 4:** Spearman’s correlation coefficients (r) of CTP parameters (symptomatic limb), angiographic parameters and hemodynamic parameters in all patients (n = 35) and in the occlusion group (n = 27).

*Parameters*	Blood flow		Blood volume		Mean transit time	
	*r*	*p-value*	*r*	*p-value*	*r*	*p-value*
*All patients*						
Lesion length (n = 35)	0.52	0.001	0.65	0.000	0.28	0.10
Degree of collateralization (n = 35)	-0.001	1.00	-0.11	0.54	-0.05	0.80
ABI (n = 28)	-0.34	0.08	-0.56	0.002	-0.12	0.55
ABP (n = 28)	-0.22	0.26	-0.56	0.002	-0.28	0.15
SPO (n = 35)	-0.21	0.23	-0.08	0.65	0.13	0.44
PSV (n = 29)	-0.06	0.75	0.05	0.79	0.14	0.46
*Occlusion group*						
Lesion length (n = 27)	0.47	0.01	0.58	0.002	0.32	0.10
Degree of collateralization (n = 27)	0.01	0.95	-0.07	0.74	-0.05	0.81
ABI (n = 22)	0.32	0.15	-0.56	0.007	-0.20	0.38
ABP (n = 22)	-0.24	0.28	-0.58	0.005	-0.30	0.18
SPO (n = 27)	-0.18	0.37	0.01	0.98	0.20	0.22
PSV (n = 22)	0.06	0.80	0.25	0.26	0.22	0.33

ABI: ankle-brachial pressure index, ABP: ankle blood pressure, PSV: peak systolic velocity, SPO: segmental pulse oscillography.

**Fig 3:**
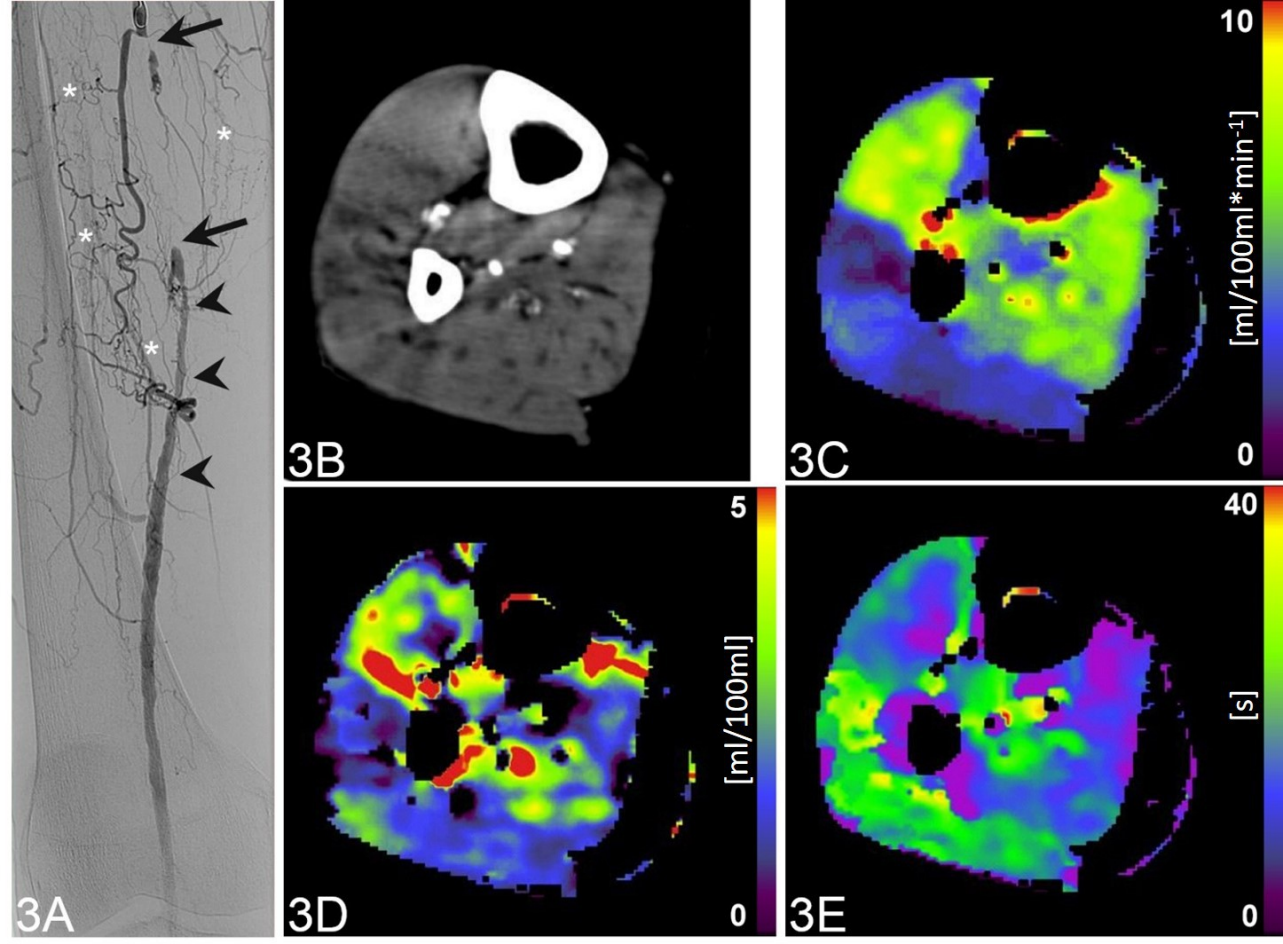
Occlusion of the right-sided superficial femoral artery of a 72-year-old patient. Coronal digital subtraction angiography image (A) shows a complete intermediate-length occlusion (arrows) of the right right-sided superficial femoral artery, which is extensively collateralized (asterisks). The distal segment of the vessel is patent, however, with caliber irregularities (arrowheads). CTP of the lower leg (B, maximum intensity projection CT image during 60s after contrast administration) with color-coded parametric maps (C-E) reveals a mean blood flow of 5.54 ml/100ml*min^-1^ (C), mean blood volume of 0.81 ml/100ml (D) and mean transit time of 12.85 s (E).

No significant correlation was found between CTP parameters and the degree of collateralization, SPO and PSV. A patient with occlusion of the right-sided superficial femoral artery is shown in **Fig 3.**

## Discussion

Our study shows that semiquantitative CTP imaging of the lower limb is feasible. To our knowledge, this is one of the earliest reports evaluating semiquantitative CTP values of the lower leg in PAD patients. Our study analyses the correlation of CTP parameters with state-of-the-art clinical parameters and with invasive angiographic assessment.

We could show that BF and BV are higher in subjects with longer lesions, and that BV is higher in subjects with lower ABI. In the subgroup with short lesions, BF and BV were lower compared to the asymptomatic limb. In our collective, patients with medium and long lesions had significantly better collateralization than patients with short lesions. Furthermore, we found a positive correlation between CTP parameters BF and BV and the lesion length, and an inverse correlation between BV and ABI. No significant correlation was found between CTP parameters and SPO or PSV.

The ABI is a well-established indicator for PAD, which is generally used for clinical evaluation of symptomatic patients. Such was true in our study cohort as well, since only one of 28 patients (3.6%) without suspicion of media sclerosis had a normal ABI value [31]. However, ABI is a global measure of perfusion and does not account for regional differences in the leg [13]. Because treatment options in PAD patients are still limited, the ABI is not recommended as a general screening test [32]. This might change in the future, since several new molecular and regenerative therapeutic agents are under investigation [32, 33]. However, there is a basic need for a method that provides reliable, reproducible, and observer-independent quantitative assessment of extremity perfusion. Such a method ideally reflects the entire extent of disease, and not only the morphological surrogate which is partly responsible for the acute symptoms of patients. Furthermore, such a method should not be solely based upon the blood flow in the largest vessels of the leg, which is the basic underlying physiologic parameter of the ABI. Being able to provide better diagnostic overview would potentially allow to more accurately stratify the collective of treatable patients, optimally with therapeutic options at hand in early disease stages.

In patients with a pathologic ABI, further diagnostic tests are necessary in order to localize lesions and assess the grade of stenosis. Several different modalities provide detailed characteristics of the clinically relevant lesion. To date, invasive diagnostic tests are the first line recommendation for patients eligible for revascularization [32]. However, the causative lesion (stenosis or occlusion) and the acute symptoms do neither reflect the whole extent of the disease, the degree of functional (and not only morphological) collateralization, and the consecutive compensatory mechanisms nor do they provide information on hypoxic downstream tissue at risk. In our study, ABI values were even inversely correlated to the BV of downstream musculature. Besides proximal vessel pathology, other factors such as chronic downstream microvascular occlusion are limiting the lifetime of the dependent musculature as well. Such was shown repeatedly in well investigated cardiovascular patients with coronary artery disease and might be one of the reasons that coronary artery stents were yet not proven to decrease the mortality. This fact has changed the diagnostic workup of coronary artery disease with non-invasive test, including contrast-enhanced CT imaging, are a first-line recommendation now, and it might change the work-up of PAD as well. The first reason is that the invasive angiography bears an inherent risk of complications, although in peripheral arteries possible complications might be manageable more easily compared to cardiac and CNS angiography. Another reason is that the interventional angiography, as well as all other currently used assessments such as ABI, ABP, SPO, and PSV does not account for microvascular disease in a quantitative manner. Furthermore, these modalities hardly consider the collateralization of vessels or neo-angiogenesis. On the other hand, CTP parameters were shown to reflect microvessel density in tumours and might serve as a more throughout assessment tool of vascular disease compared to other modalities, including invasive tests [34]. This might open up the way for early first line assessment of PAD and for response assessment to modern molecular treatment options.

In a CTP study by Barfett et al., a significant difference of foot perfusion values between the side of simulated vascular obstruction and the non-obstructed side were shown [13]. In our study, this was true only in the subgroup of patients with short lesions, where BF and BV were lower compared to the asymptomatic side. In the other patients, the pre-interventional CTP values of the symptomatic limb were not significantly different to the asymptomatic side. The reason might be that in longer lesions, the vascular bed has had more time to develop sufficient collaterals. In line with this reason is the fact, that in our study BF and BV were higher in limbs with long lesions, compared to those with short lesions, and that BV and BF correlated with lesion length. This collateralization leads then to a successive diminution of the collateralized vessel–until another acute event occurs, either proximal or distal to the collateralized segment, which then overburdens the collateral capacity. This factor was not studied by Barfett et al., since their study was performed in healthy subjects. With regard to our results (BF and BV differences in symptomatic and asymptomatic limbs, less collaterals with short lesions), CTP might be particularly useful in patients with short lesions (or early PAD) where there is a relative lack of collateralization.

Since PAD is considered a generalized disease, changes of the microvascular density might alter the vascular supply of the muscles significantly on both the symptomatic and the asymptomatic side. On one hand, the microvascular occlusion might reduce the perfusion of the muscles. On the other hand, it has been shown that in limbs with a severe stenosis, ischemia induces neo-angiogenesis [35]. This may be reflected in higher than expected perfusion values in a limb with a hemodynamically relevant stenosis compared to the other side, where only microvascular disease is found. Another possible reason for the similar CTP values of symptomatic and asymptomatic limb in our patients with long lesions might be that the scans were acquired during a rest situation. Such is well known from myocardial perfusion examinations, where ischemic myocardium typically appears normal at rest. Stress conditions, which are known to provoke clinical PAD symptoms, were not simulated in our study. Due to disease-specific alterations of the vessel wall, e.g. calcifications, the consecutive lack of elasticity might prevent the vessels from physiologic contraction under rest situations. This should be investigated in future studies comparing the perfusion at rest and during stress, in order to compare the “functional flow reserve” of symptomatic and asymptomatic limbs.

The assessment of CT perfusion in calf muscles, as performed in our study, might have advantages over the perfusion measurement in the skin or muscles of the foot. We expect perfusion values obtained in muscle tissue be more reliable compared to skin-derived values, since they are more independent of ambient temperature and resistant to movement artefacts if patients are positioned properly [12, 13, 16]. However, further studies on state-of-the-art scanners are necessary to compare perfusion measurements at different levels of the leg intra-individually. Recapitulating our results, we have shown that CTP values obtained in calf musculature, which represents the anatomic substrate for the clinical hallmark of critical limb ischemia, i.e. claudication, might reflect an appropriate overview of the local (limb related) PAD. CTP takes into account the functional collateralization and physiological compensation mechanisms of the body. It might therefore be an appropriate tool for PAD patients, complementing the diagnostic workup. Furthermore, CTP scans with a larger craniocaudal field-of-view may cover the majority of the leg, and when comparing perfusion values at different levels might help predict the appropriate level for intervention with regard to the emerging concept of angiosome-specific treatment [34–36].

Besides stenosis diameter and length, BF is also determined by partly microscopic parameters that are more difficult to quantify exactly, such as microvascular obstruction and collateralization. Our study cannot identify which one of these parameters is the dominant factor determining BF. This might be the reason for not finding a strong correlation between CTP and different parameters.

It should be mentioned that our study has some limitations. We did perform consensus reading instead interreader evaluation of results [37]. There are several software with different underlying calculation models available and evaluation of the reading performance with different software/models was beyond the scope of our initial evaluation [38, 39]. Also, we do not provide CTP values of a collective of healthy subjects for comparison. While this is a major drawback, it is ethically hard to justify obtaining CTP data from healthy volunteers. Those would have to be age-matched, too (and any elderly patient might not be considered as “vascular healthy” anymore). Such healthy volunteer scanning might be justifiable in large patient collectives, however not necessarily in an initial evaluation like ours.

Asymptomatic limbs in our cohort are inherent parts of the systemic disease as well, and as shown in our study, cannot serve as standard of reference for absolute CTP values. Future work might analyse CTP parameters in patients with critical limb ischemia at rest and at stress, and before and after revascularization. Also, we did not compare CTP with other quantifiable perfusion imaging, such as PET perfusion. Although an accurate technique, it is only available in a few centers worldwide, owing to financial, technical and logistic issues [40, 41]. Finally, CTP parameters were overall not significantly different in the symptomatic and asymptomatic limb. The main reason is probably that our study was acquired at rest, not at stress. Another reason might be the relatively small sample size. Furthermore, since PAD is considered a generalized disease, changes of the microvascular density might alter the vascular supply of the muscles significantly in both the symptomatic and the asymptomatic side. On one hand, the microvascular occlusion might reduce the perfusion of the muscles. On the other hand, it has been shown that in limbs with a severe stenosis (and subsequent hypoxia), ischemia induces neoangiogenesis.

Overall, since there was a difference in CTP parameters in patients with short stenosis, one possible explanation might be that using CTP-values in direct intra-individual comparison in this indication works best in a specific time window (e.g. after initial/early vessel narrowing).

## Conclusions

In our study we showed that BF and BV are higher in subjects with longer lesions and that BV is higher in subjects with lower ABI. In a subgroup with short lesions, BF and BV were lower compared to the asymptomatic limb. Furthermore, we found a positive correlation between CTP parameters BF and BV and the lesion length, and an inverse correlation between BV and ABI. In the future, this quantitative method might serve as a non-invasive method, which might complement the diagnostic workup of patients with peripheral arterial disease.

## Supporting information

S1 File Further details of recorded data, inclusion criteria and hemodynamic assessment are given in Supporting information File. (DOCX)

S1 Table Mean of length of lesion (mm) for the different groups of collateralisation. Patients with medium and long lesions had a higher degree of collateralization than patients with short lesions, (p = 0.041).
(DOCX)

## Abbreviations

3D: Three-dimensional
4D: Four-dimensional
ABI: Ankle-brachial pressure index
ABP: Ankle blood pressure
BF: Blood flow
BV: Blood volume
CM: Contrast medium
CT: Computed tomography
CTP: CT-perfusion
CW: Continuous wave
HU: Hounsfield unit
MIP: Maximum intensity projection
MRI: Magnetic resonance imaging
MTT: Mean transit time
PACS: Picture archiving and communication system
PAD: Peripheral arterial disease
PSV: Peak systolic velocity
*r*: Correlation coefficient
r^2^: Correlation coefficient of determination
ROI: Region of interest
SD: Standard deviation
SPO: Segmental pulse oscillography
US: Ultrasound
VOI: Volume of interest
