# Supplementary material for: CT-perfusion in peripheral arterial disease – Correlation with angiographic and hemodynamic parameters
Source: PLoS One. 2019 Sep 27;14(9):e0223066. doi: 10.1371/journal.pone.0223066 (PMC6764684; doi:10.1371/journal.pone.0223066)
Supplement: S1 File — (DOCX) [file pone.0223066.s001.docx]

**Supporting information**

**S1 File:**

A medical history was recorded, comprising of the aforementioned symptoms as well as prior surgical revascularization and catheter revascularization interventions. Current medications were recorded as well as a history and symptoms of allergic reactions. This was routinely followed by a physical examination consisting of the evaluation of clinical signs of peripheral arterial disease, such as decreased temperature and pale colour of the skin, diminished or absent pulses from the abdominal aorta to the ankles, bruits and trophic lesions.

**Exclusion criteria were**: renal insufficiency (renal clearance below 30 mL/min) without dialysis, known allergy or hypersensitivity to iodinated CM, untreated hyperthyroidism, pregnancy.

**Hemodynamic Assessment**

ABI: The systolic pressure of the posterior tibial and dorsal pedal artery was measured using appropriately sized blood pressure cuffs placed around the calf just above the ankles. The systolic pressure of the dorsal pedal artery and the posterior tibial artery were measured at the dorsum pedis and between the medial malleolus and the Achilles tendon respectively by detecting the first typical signal with an 8 MHz continuous wave (CW) Doppler probe during slow deflation of the cuffs after inflation to suprasystolic levels.

Ultrasonographic imaging: The location and distribution of lesions including occlusions was documented using a standardized form. In subjects with vessel stenosis, the ratios of peak systolic velocity (PSV; m/s) in the stenotic segment and prestenotic PSV were calculated. Other parameters such as turbulent flow were also used to quantify the degree of stenotic lesions. No flow with colour-coded and pulsed wave Doppler imaging indicated occlusion of a given arterial segment.
