# Supplementary material for: CT-perfusion in peripheral arterial disease – Correlation with angiographic and hemodynamic parameters
Source: PLoS One. 2019 Sep 27;14(9):e0223066. doi: 10.1371/journal.pone.0223066 (PMC6764684; doi:10.1371/journal.pone.0223066)
Supplement: S1 Table — Patients with medium and long lesions had a higher degree of collateralization than patients with short lesions, (p = 0.041). (DOCX) [file pone.0223066.s002.docx]

**S1 Table:** Mean of length of lesion (mm) for the different groups of collateralisation. Patients with medium and long lesions had a higher degree of collateralization than patients with short lesions, (p = 0.041).

| **Collateralization** | **n** | **Mean** | **SD** |
| --- | --- | --- | --- |
| 1 | 11 | 11.46 | 8.12 |
| 2 | 17 | 8.82 | 7.69 |
| 3 | 7 | 8.43 | 8.4 |
| All | 35 | 9.57 | 7.83 |
